# The Enhanced Immune Protection in Small Abalone *Haliotis diversicolor* Against a Secondary Infection With *Vibrio harveyi*

**DOI:** 10.3389/fimmu.2021.685896

**Published:** 2021-07-06

**Authors:** Tuo Yao, Jie Lu, Changming Bai, Zhilv Xie, Lingtong Ye

**Affiliations:** ^1^ Key Laboratory of South China Sea Fishery Resources Exploitation & Utilization, Ministry of Agriculture and Rural Affairs, South China Sea Fisheries Research Institute, Chinese Academy of Fishery Sciences, Guangzhou, China; ^2^ Key Laboratory of Maricultural Organism Disease Control, Ministry of Agriculture and Rural Affairs, Qingdao Key Laboratory of Mariculture Epidemiology and Biosecurity, Yellow Sea Fisheries Research Institute, Chinese Academy of Fishery Sciences, Qingdao, China; ^3^ Key Laboratory of Aquatic Product Processing, Ministry of Agriculture and Rural Affairs, South China Sea Fisheries Research Institute, Chinese Academy of Fishery Sciences, Guangzhou, China

**Keywords:** *Haliotis diversicolor*, *Vibrio harveyi*, enhanced immune protection, secondary infection, immune priming

## Abstract

In recent years, more and more studies have shown that early pathogenic bacterial infection in invertebrates can enhance immunity and significantly reduce mortality when reinfected with the same pathogen. There are mechanisms to explain this phenomenon, but they are relatively few. In addition, dose-dependent primary infection is also associated with increased immunity. In the present study, the initial infection dose and mortality of abalone *Haliotis diversicolor* after reinfection with *Vibrio harveyi* were recorded, and the mechanism of immune enhancement was investigated by the transcriptomic response of abalone after two successive stimuli with *V. harveyi*. Priming with different concentrations of pathogen can enhance immunity; however, higher concentration is not always better. Compared with the first exposure, more genes were up-regulated after the second exposure. Among the commonly expressed genes, the immune related genes were significantly or persistently highly expressed after two infections and included pattern recognition receptors as well as immune effectors, such as toll-like receptors, perlucin 4, scavenger receptor class B-like protein, cytochrome P450 1B1-like, glutathione S-transferase 6, lysozyme and so on; in addition, these immune-related genes were mainly distributed in the pathways related to phagocytosis and calcium signaling. Among the specifically expressed genes, compared with the first infection, more genes were involved in the immune, metabolic and digestive pathways after the second infection, which would be more conducive to preventing the invasion of pathogens. This study outlined the mechanism of immune enhancement in abalone after secondary infection at the global molecular level, which is helpful for a comprehensive understanding of the mechanism of immune priming in invertebrates.

## Introduction

More and more studies have shown that the innate immune system has a memory similar to the adaptive immune system, which endows the organism with a stronger and more effective resistance to reinfection, and can be found in a variety of organisms (1, 2). This characteristic is found in plants (3), bacteria (4, 5) and viruses (6). And, this phenomenon is defined in different terms depending on the species studied, for example “trained immunity” in vertebrates (7–9), “immune priming” in invertebrates (10, 11) and “Systemic Acquired Resistance” (SAR) in plants (12, 13). Based on molecular, immunological and evolutionary arguments, Netea et al. (9) proposed that innate immune memory is a primitive form of immune memory, while adaptive immune memory is an advanced form of immune memory. It is necessary to develop an immune memory as it is of great advantage in improving the organism’s survival rate in an unfavorable environment.

Invertebrates include a wide variety of species, accounting for more than 95% of the animal kingdom (14). Invertebrate immunology has attracted more and more attention, and with the further study of invertebrate immune priming, some mechanisms have been identified. It is generally believed that improved resistance after reinfection is mainly related to the up-regulated expression of pattern recognition receptors (PRRs) and the enhancement of phagocytic activity. For example, fibrinogen-related proteins (FREPs) were over represented in the Vector Snail following a secondary challenge (15). Following re-exposure to the pathogen, C-lectins and peptidoglycan recognition protein-S1 were up-regulated in scallop (16, 17). Increased phagocytosis was found in silkworm (18), Drosophila (19) and Pacific oyster (20) after homologous exposure. In addition, other mechanisms such as DNA synthesis (21) and RNA methylation (22) have also been used to explain immune priming. Moreover, initial priming doses can affect the immune priming outcome. A positive correlation between increased resistance and priming dose was found in *Galleria mellonella* larvae (23). However, immune priming is not universal. Immune memory failed to be detected in damselflies (24) and ants (25).

Abalone is an important economic shellfish in China, and plays a pivotal role in the aquaculture of marine shellfish. Compared with 2018, production in 2019 increased by 9.85%; the annual growth rate far exceeds that of other farmed shellfish. Although abalone farming is generally on the rise in China, the frequent occurrence of disease has seriously affected the rapid development of abalone aquaculture. The cultivation and promotion of new varieties of abalone have promoted the revitalization and development of the abalone breeding industry to a certain extent, but diseases still occur from time to time 3–5 years after the breeding of new varieties. *Vibrio harveyi* is a Gram-negative bacterium, which is widely distributed in various waters and has high pathogenicity in abalone. In previous studies, we confirmed that improved survival rate could be obtained during re-infection when *Haliotis diversicolor* was primed with *V. harveyi*. However, it is not clear whether the initial priming dose is associated with increased resistance in re-infection. Although mechanisms of immune priming at the cytological level have been studied in abalone (26), molecular studies have not been reported. To fully understand the basis of innate immune memory generation, a global molecular approach is needed. The development of high-throughput sequencing technology has accelerated the study of non-model organisms and made it possible to investigate immune priming mechanisms at the overall molecular level.

In order to determine whether different infection doses affect resistance and address the mechanism of immune enhancement after abalone secondary infection, in this study, two different experiments were conducted. In the first experiment, *H. diversicolor* were exposed to different concentrations of *V. harveyi* in the first infection. However, the same concentration was used in the second infection to detect the relationship between the infection dose and immune resistance *via* survival rate changes. In the second experiment, we examined the alterations in mRNA expression of immune-related genes during one and two infections of abalone, respectively, using a transcriptomic approach. In this study, we hoped to understand the mechanism of improved immunity during re-infection.

## Materials and Methods

### Abalone and Microbes

The abalone (mean shell length 41.35 ± 0.23 mm) used in this study were obtained from our aquaculture base in Jieyang, Guangdong Province, China. The animals were acclimated in experimental barrels with continuous oxygenation and a flow-through sea water supply for 2 weeks. Filtered seawater and salinity were maintained at 28°C and 30, respectively.

*V. harveyi* isolated from the hepatopancreas of moribund abalone, was used in these experiments. The bacteria were incubated in LB medium at 28°C for 20 h, and harvested by centrifugation at 7,000 × *g* at 25°C for 5 min. The pellet was resuspended in sterile sea water (SSW) which was filtered using a 0.2 μm Millipore filter, and the final concentration was adjusted according to experimental requirements.

### Immune Challenge and Sample Collection

**Figure 1:**
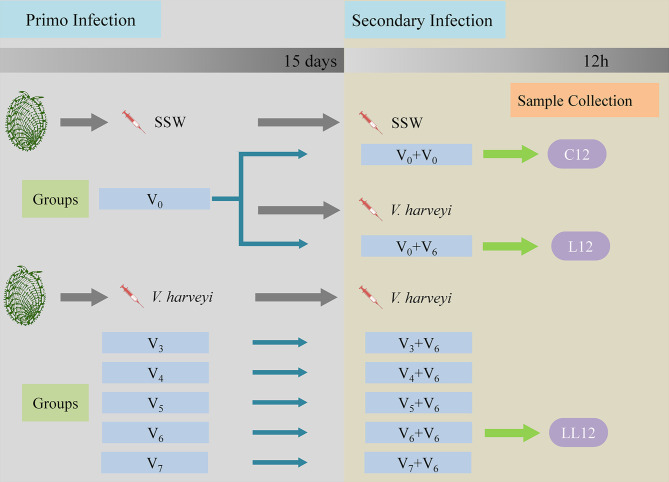
Schematic diagram of the experimental design. Each experiment consisted of two successive infection challenges. For the primary immune challenge, abalone from the same batch were injected with either sterile sea water (SSW, group V_0_) or different concentrations of *V. harveyi*: 1.42 × 10^3^ CFU mL^-1^, 1.42 × 10^4^ CFU mL^-1^, 1.42 × 10^5^ CFU mL^-1^, 1.42 × 10^6^ CFU mL^-1^ and 1.42 × 10^7^ CFU mL^-1^, designated as V_3_, V_4_, V_5_, V_6_ and V_7_, respectively. Fifteen days later, the five treatment groups and the V_0_ group received an injection of *V. harveyi* at a concentration of 1.58 × 10^6^ CFU mL^-1^ (designated as V_3 + 6_, V_4 + 6_, V_5 + 6_, V_6 + 6_ V_7 + 6_ and V_0 + 6_). The V_0_ group was also injected with SSW as a control for the secondary infection (V_0 + 0_). During each experimental infection, mortalities were monitored daily post-challenge. The abalone hepatopancreases were sampled 12 h after the second stimulation in the V_0 + 0_, V_0 + 6_ and V_6 + 6_ groups, and named C12, L12 and LL12 groups, respectively. Gene expression levels in the hepatopancreas were then analyzed by transcriptomics.

For observation of immune priming, the abalone received two immune challenges in total. A schematic diagram of the experimental design is shown in **Figure 1**. For the primary immune challenge, abalone were divided into five treatment groups designated as V_3_, V_4_, V_5_, V_6_ and V_7_, and were injected with 50 μL of different concentrations of *V. harveyi* as follows: 1.42 × 10^3^ CFU mL^-1^, 1.42 × 10^4^ CFU mL^-1^, 1.42 × 10^5^ CFU mL^-1^, 1.42 × 10^6^ CFU mL^-1^ and 1.42 × 10^7^ CFU mL^-1^, respectively. Abalone in a control group received an injection of 50 μL SSW as a control (designated the V_0_ group). Fifteen days later, the secondary immune challenge was performed. Five treatment groups designated as V_3 + 6_, V_4 + 6_, V_5 + 6_, V_6 + 6_ and V_7 + 6_, corresponding to the five primary immune challenge groups were each injected with 50 μL *V. harveyi* at a concentration of 1.58 × 10^6^ CFU mL^-1^ for the secondary immune challenge. In addition, the SSW group was divided into two subgroups, designated as V_0 + 0_ (receiving an injection of 50 μL SSW) and V_0 + 6_ (receiving an injection of 50 μL *V. harveyi* at the concentration of 1.58 × 10^6^ CFU mL^-1^). During the first injection, 360 abalone were used in each group. During the second infection, 90 abalone in each group were used for mortality monitoring, and at least 30 abalone were used in each of the sampling groups. All experiments were performed in triplicate with abalone in 500 L plastic tanks. In these experiments, dead animals were removed in a timely manner to avoid affecting the water quality.

To study the correlation between initial infection dose and degree of protection, daily mortality rates in each group were monitored. To study the molecular mechanism of immune priming, the hepatopancreases of live abalone were randomly sampled at the time point of 12 h after the second stimulation from the V_0 + 0_, V_0 + 6_ and V_6 + 6_ groups, and were named the C12, L12 and LL12 groups, respectively. A total of nine abalone were sacrificed in each group, and three hepatopancreases from each replicate were pooled together as one sample. These samples were immediately frozen in liquid nitrogen and stored separately at -80°C for subsequent transcriptome sequencing. The survival rate was calculated with a Kaplan-Meier estimate followed by a log-rank test in SPSS 25. Significant differences were set at *P* < 0.05.

### RNA Extraction, Library Construction, and Sequencing

Total RNA was extracted using the TRIzol kit (Invitrogen, Carlsbad, CA, USA) according to the manufacturer’s instructions. Total RNA samples were then digested with DNase I (Ambion, Thermo Scientific, Waltham, MA, USA) to remove potential genomic DNA contamination. RNA degradation and contamination were monitored in 1% agarose gels. RNA quantity and integrity were measured using a NanoDrop 2000 spectrophotometer (ThermoFisher Scientific, Wilmington, DE, USA) and an Agilent 2100 Bioanalyzer (Agilent Technologies, Santa Clara, CA, USA), respectively. The resultant RNA samples were then used for RNA-seq.

The poly (A) mRNA was enriched using poly-T oligo-attached magnetic beads (Dynabeads; Invitrogen, Carlsbad, CA, USA) and then cleaved into fragments in the NEB proprietary fragmentation buffer. Following first strand and complementary strand synthesis, the resultant RNA was ligated with sequencing adapters. Then PCR amplification was performed to generate the RNA-seq library. The library preparations were sequenced on an Illumina Novaseq 6000 platform (Illumina, San Diego, CA, USA) and 150 bp paired-end reads were generated.

### Bioinformatics Analysis

Clean reads were obtained from the raw reads (149,598,910, 148,942,646 and 165,147,008 reads in C12, L12 and LL12 groups, respectively) by removing adapter reads, unknown reads (with ‘N’ ratios > 10%), low quality reads (with quality value ≤ 20) and short reads (with length < 30 bp). After this processing, 148,180,604, 147,780,808 and 163,874,166 clean reads were obtained from the C12, L12 and LL12 groups, respectively (**Table 1**). The resultant reads were then assembled into a transcriptome using Trinity software with default settings (27). BlastX was used to obtain functional annotation of all expressed genes, by comparing with six databases, including the NCBI nonredundant protein (NR), Swiss-Prot, Protein family (Pfam), Gene Ontology (GO), Cluster of Orthologous Groups (COG) and Kyoto Encyclopedia of Genes and Genome (KEGG) databases, with a cut-off E-value of < 10^-5^. GO annotations were analyzed with the Blast2GO program (28) utilizing default parameters. After treatment, the mapping rates ranged from 56.34% to 59.68% for all groups (**Table 1**). The expression abundance of *H. diversicolor* was calculated using RSEM software (29) and gene expression levels were measured using the FPKM (Fragments Per Kilobase of transcript per Million fragments) method. Differentially expressed genes (DEGs) in the different groups were detected using the DESeq package (30). Significant differential expression was defined by setting absolute log_2_Flodchange > 1 and FDR (false discovery rate) p-value (q-value) < 0.05 as the threshold. Finally, the obtained DEGs were included in the GO and KEGG enrichment analysis, based on the Hypergeometric distribution model.

**Table 1:** Summary of the transcriptome assembly.

Sample	Raw reads (×10^6^)	Raw bases (×10^8^)	Clean reads (×10^6^)	Clean bases (×10^8^)	Q20 (%)	Q30 (%)	GC content (%)	Mapped reads (×10^6^)	Mapped ratio (%)
C12_1	44.55	67.26	44.08	65.43	98.82	95.98	45.64	25.86	58.67
C12_2	51.53	77.81	51.04	75.54	98.88	96.15	46.09	29.82	58.42
C12_3	53.52	80.82	53.07	78.82	98.95	96.35	46.60	31.67	59.68
L12_1	46.74	70.58	46.31	68.90	98.86	96.13	46.30	26.09	56.34
L12_2	50.08	75.62	49.72	73.88	98.99	96.45	45.30	29.00	58.33
L12_3	52.12	78.71	51.76	76.99	98.93	96.28	45.76	29.86	57.69
LL12_1	55.36	83.59	54.93	81.53	98.93	96.27	45.85	32.55	59.26
LL12_2	56.45	85.24	56.03	83.13	98.98	96.44	46.13	32.18	57.43
LL12_3	53.34	80.55	52.91	78.53	98.96	96.41	46.30	30.12	56.93

### Gene Expression Validation

Twelve DEGs, including toll-like receptor (TLR), TLR2, TLR8, scavenger receptor class B-like protein (SR‐BI), X-box binding protein (XBP), cathepsin B (CatB), tumor necrosis factor ligand superfamily member 6-like (TNFSF6), legumain (Lgmn), Zinc transporter ZIP10 (ZIP10), peptidoglycan-recognition protein SC2 isoform X1 (PGRP-SC2), pannexin 5 (Px5) and tumor necrosis factor ligand superfamily member 6 isoform X2 (TNFSF6-X2) were selected to validate Illumina sequencing data by real-time qRT-PCR analysis, and qPCR was performed on different individuals exposed to the same conditions. All primers were acquired with Primer Premier 5.0 based on the reference transcriptome sequences and β-actin was selected as the reference gene (**Table 2**). RNA preparation was carried out as above. The first strand cDNA was synthesized from approximately 1 μg of total RNA using the PrimeScript™ RT Reagent Kit (Takara, Dalian, China) in a 20 μL reaction system following the manufacturer’s protocol. The qRT-PCR amplifications were performed with a LightCycler^®^ 480 system (Roche Biochemicals, Indianapolis, IN, USA) using TB Green^®^ Premix Ex Taq™ II (Takara, Dalian, China) in a 10 μL reaction system. The thermal cycling parameters were 95°C for 30 s, followed by 40 cycles of 95°C for 5 s and 60°C for 30 s. Three biological replicates and three technical replicates were included in these experiments and the data were obtained using the 2^−ΔΔCT^ method (31). The data were expressed as the mean ± SD (n = 3).

**Table 2:** Sequences of the primers used in this study.

Primer	Primer sequence (5’-3’)
β-actin-F	CCGTGACCTTACAGACTACCT
β-actin-R	TACCAGCGGATTCCATAC
TLR-F	CCTCAAAGAACGGTCCCA
TLR-R	CGTCAGGCAGAGCGAAA
TLR8-F	CCACCAGCGAGACTTTGC
TLR8-R	CTGTGCGGAACTCCATCA
SR-BI-F	CTATTCTTACAGGGAGCATCG
SR-BI-R	CGCTGAAACTCAAACCACC
TLR2-F	ACACAAAGCAAGGGTCAA
TLR2-R	TCAACAGCGTGGAGGAT
XBP-F	AGAGGGGCGTATTCAGA
XBP-R	GCCATTGGTCGGGTGTA
CatB-F	GTGGAAGGCTGGTAGAAACG
CatB-R	CATTGATGTCCTTTACACCCA
TNFSF6-F	CCAGACACCGCTGAGAATG
TNFSF6-R	GGACAATCAATACCGCAAATA
Lgmn-F	ATGGACAAGGTGCGAAAG
Lgmn-R	CCCTCCTGACAATCTCAAACT
ZIP10-F	GGCAAGCAAGAACCAAG
ZIP10-R	CCATTTCCCCTATGACCTG
PGRP-SC2-F	CCTCATTCCATCAGCCATCT
PGRP-SC2-R	CCTATCCTGTCCCAGCCAC
Px5-F	CCGAAAGAATACGACAAGG
Px5-R	GATGACCCAACGGTAGAAG
TNFSF6-X2-F	GGGCGGATTGACTTTGC
TNFSF6-X2-R	ATTCGGTTGTCTTGGATGTT

## Results

### Survival Rates After First and Second Challenge With *V. harveyi*

**Figure 2:**
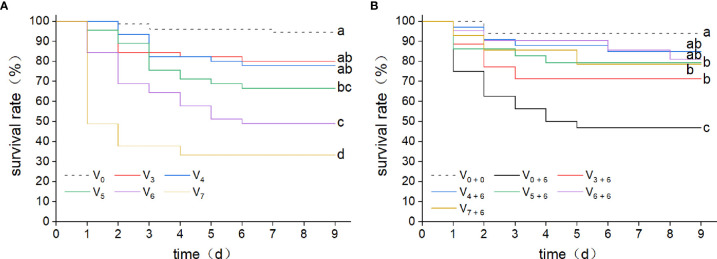
Kaplan-Meier survival curves for abalone after two consecutive infections with *V. harveyi*. **(A)** Kaplan-Meier survival curves were generated from abalone following a first injection with *V. harveyi* at concentrations of either 1.42 × 10^3^ CFU mL^-1^ (V_3_), 1.42 × 10^4^ CFU mL^-1^ (V_4_), 1.42 × 10^5^ CFU mL^-1^ (V_5_), 1.42 × 10^6^ CFU mL^-1^ (V_6_) or 1.42 × 10^7^ CFU mL^-1^ (V_7_), or with SSW as control (V_0_). A total of 360 abalone were used in each group (120 per tank). Mortalities were monitored for nine days after infection. Different letter labels next to the graph lines indicate statistically significant differences among treatments (*p* < 0.05, log-rank test, n = 360). **(B)** Kaplan-Meier survival curves were generated from abalone primed by injection with *V. harveyi* at five different concentrations, or with SSW, followed by a second injection with *V. harveyi* at 1.58 × 10^6^ CFU mL^-1^ (V_3 + 6_, V_4 + 6_, V_5 + 6_, V_6 + 6_ V_7 + 6_ and V_0 + 6_) or with a second SSW injection as control (V_0 + 0_). A total of 90 abalone were used in each group (30 per tank). Mortalities were monitored for nine days after infection. Different letter labels next to the graph lines indicate statistically significant differences among treatments (*p* < 0.05, log-rank test, n = 90).

To investigate the relationship between immune protection and initial dose of infection, two consecutive *V. harveyi* infection experiments were performed. In this study, five different concentrations of *V. harveyi* diluents were used. After the first immune stimulation, the survival rate decreased with increased infection concentration (**Figure 2A**). Among them, the survival rate in the V_6_ group was 48.9%. Thus, a bacterial concentration of 10^6^ CFU mL^-1^ was selected for the second infection experiment. After the second infection, the log-rank test showed that the survival rates in all groups receiving the first immune stimulation were significantly higher than that in group V_0 + 6_ (**Figure 2B**), but there were no significant differences among groups V_3 + 6_, V_4 + 6_, V_5 + 6_, V_6 + 6_ and V_7 + 6_ (*P <* 0.05). This implies that, the previous infection protected the abalone against a secondary infection. However, there was no positive correlation between immune protection and the initial dose of infection, as group V_7+6_ did not have the highest survival rate.

### Analysis of Sequenced Data Quality

Nine cDNA libraries were constructed for Illumina sequencing and the data processing results are summarized in **Table 1**. After assembly, the length of these transcripts ranged from 201 to 16,691 bp with an average length of 885 bp, and the N50 length was 1,311 bp. The values of the Q30 were all > 95.9% and the GC percentage of the clean reads in the nine libraries ranged from 45.30% to 46.60%, suggesting a good assembled quality and sufficient for subsequent analysis. Raw sequencing data were archived under the accession ID SRR13931757-SRR13931765 in the NCBI Sequence Read Archive.

### Cluster Analysis and Pairwise Comparisons

**Figure 3:**
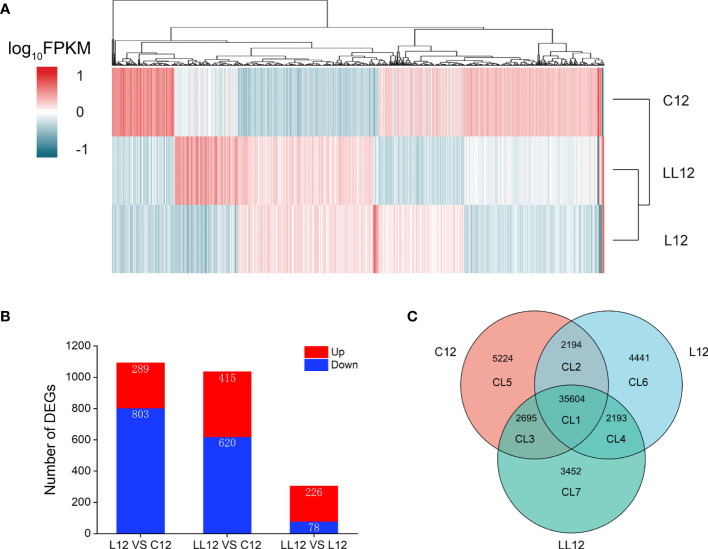
Overview of the expression analysis for C12, L12 and LL12. **(A)** Hierarchical clustering heat map constructed based on DEGs. Each row represents a group, each column represents a DEG. The expression level of each DEG is shown as the log_10_(FPKM) value. Red and blue gradients indicate increased and decreased transcript abundance, respectively. **(B)** Number of DEGs in the different groups. **(C)** Venn diagram showing the number of commonly and uniquely expressed genes among different groups.

In order to comprehensively understand the distribution of different genes, a hierarchical cluster analysis was performed for all DEGs (**Supplementary File 1**). As shown in **Figure 3A**, the stimulus group sample, including L12 and LL12, formed one cluster, and were then grouped with C12. When compared with C12, 1092 DEGs were identified in L12 (289 up-regulated and 803 down-regulated genes) and 1,035 DEGs were identified in LL12 (415 up-regulated and 620 down-regulated genes). In addition, 304 DEGs were detected between L12 and LL12, including 226 up-regulated and 78 down-regulated genes (**Figure 3B**).

### Venn Analysis of Expressed Genes Among Different Groups

In order to better study the DEGs, a Venn diagram was constructed base on unigenes of different groups, and 7 clusters (CLs) were generated (**Figure 3C**). CL1 showed co-expressed genes in all groups. CL2, CL3 and CL4 showed co-expressed genes between groups. CL5, CL6 and CL7 showed specific expressed genes in each group. DEGs were identified in CL1, CL2, CL3 and CL4. A total of 1174 DEGs were identified in CL1 and they were further studied (**Supplementary File 2**). In CL2, four nonimmune related DEGs were identified. Thirty DEGs were generated in CL3; however, only one was an immune-related gene; thus, no further analysis was performed. Finally, no DEGs were found in CL4.

### Patterns of DEGs in CL1

**Figure 4:**
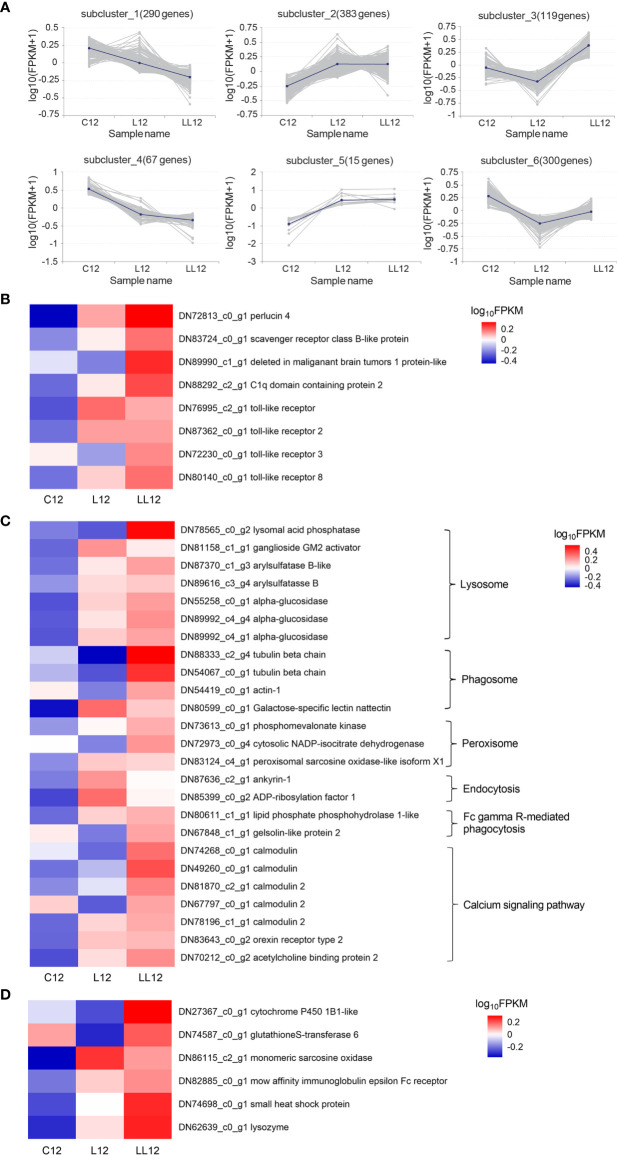
Analysis of differentially expressed genes. **(A)** Patterns of DEGs in CL1 by K-means clustering analysis. The lines represent the expression tendency of DEGs. The number of genes represented by each pattern is shown above the graphs. **(B)** The DEGs involved in the PRRs; **(C)** The DEGs involved in the immune-related KEGG. **(D)** The DEGs involved in the immune effectors. The heatmap shows log_10_ (FPKM) values of DEGs among different groups.

The DEGs in CL1 from two consecutive *V. harveyi* infections were divided into 6 subclusters based on a K-means clustering algorithm. As shown in **Figure 4A**, similar patterns in gene expression were clustered together. In subcluster 1 and 4, genes were continuously down-regulated after the first and second infection. In subcluster 6, genes decreased after the first infection and increased after the second infection; however, the expression level was lower than that in the control group. Subcluster 2 and 5 represented a class of genes that were persistently highly expressed. In subcluster 3, genes were down-regulated after one infection; however, in the second infection, the expression level increased and exceeded the control group. In view of the expression pattern, the genes in subclasses 2, 3 and 5 appeared to be the most promising possible sources of immune priming, and they were analyzed in depth.

### Immune Priming Related Genes in CL1

PRRs can bind to pathogen-associated molecular patterns (PAMPs) and are thought to be able to establish immunological memory. Eight PRRs were identified as up-regulated after the second immune challenge, including perlucin 4, C1q domain containing protein 2, scavenger receptor class B-like protein, deleted in malignant brain tumors 1 protein-like, TLR, TLR2, TLR3 and TLR8 (**Figure 4B**). Some immune-related pathways including the calcium signaling pathway and pathways associated with pathogen clearance (lysosome, phagosome, peroxisome, and Fc gamma R-mediated phagocytosis) were also activated when subjected to a continuous stimulus (**Figure 4C**). In addition, some immune effector factors including cytochrome P450 1B1-like, glutathione S-transferase 6, monomeric sarcosine oxidase, low affinity immunoglobulin epsilon Fc receptor, and lysozyme were also increased in the second infection (**Figure 4D**).

### Pathway Analysis of CL6 and CL7

**Figure 5:**
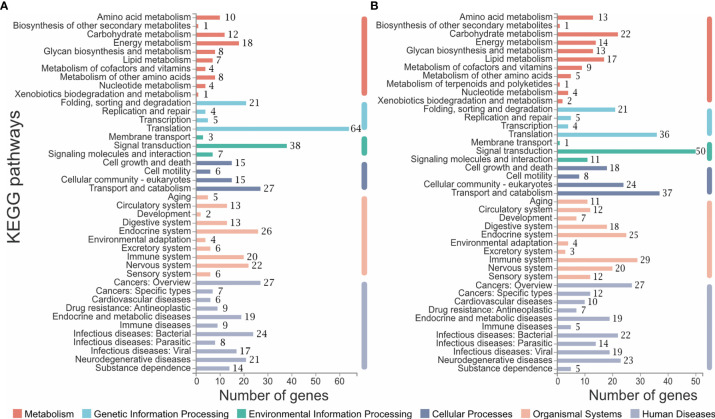
Pathway analysis of CL6 and CL7. **(A)** Pathways constituted in CL6; **(B)** Pathways constituted in CL7. The number of genes belonging to each pathway are labeled on the right of the bar.

All genes collected in CL6 and CL7 were subjected to pathway analysis, respectively. As shown in **Figure 5**, six categories of the pathways were divided into metabolism, genetic information processing, environmental information processing, cellular processes, organismal systems and human diseases. There were different gene numbers within the same pathway in CL6 and CL7. Some pathways such as carbohydrate metabolism, lipid metabolism, signal transduction, digestive system and immune system had more gene members in CL7 than in CL6. Furthermore, immune-related pathways were analyzed in depth. As shown in **Table 3**, some pathways associated with pathogen clearance had more gene numbers in CL7 than in CL6, such as lysosome, phagosome, ubiquitin-mediated proteolysis, and autophagy-animal. Other signaling pathways such as the Ras signaling pathway, NOD-like receptor signaling pathway, MAPK signaling pathway, Toll-like receptor signaling pathway and Calcium signaling pathway also showed consistent results.

**Table 3:** Gene numbers in the immune-related pathways in CL6 and CL7.

pathway ID	Description	CL6 numbers	CL7 numbers
map04142	Lysosome	9	13
map04210	Apoptosis	7	11
map04510	Focal adhesion	7	10
map04145	Phagosome	10	10
map04014	Ras signaling pathway	4	9
map04621	NOD-like receptor signaling pathway	3	8
map04010	MAPK signaling pathway	3	7
map04120	Ubiquitin mediated proteolysis	6	7
map04620	Toll-like receptor signaling pathway	1	6
map04020	Calcium signaling pathway	2	6
map04140	Autophagy - animal	2	6
map04670	Leukocyte transendothelial migration	4	6
map04068	FoxO signaling pathway	0	5
map04750	Inflammatory mediator regulation of TRP channels	3	5
map04610	Complement and coagulation cascades	0	4
map04146	Peroxisome	1	4
map04150	mTOR signaling pathway	1	4
map04664	Fc epsilon RI signaling pathway	0	3
map04650	Natural killer cell mediated cytotoxicity	1	3
map04666	Fc gamma R-mediated phagocytosis	2	2
map04062	Chemokine signaling pathway	2	2
map04144	Endocytosis	9	5
map04151	PI3K-Akt signaling pathway	7	4
map04390	Hippo signaling pathway	6	5
map04024	cAMP signaling pathway	7	6

### Validation of RNA-Seq Data by qPCR

**Figure 6:**
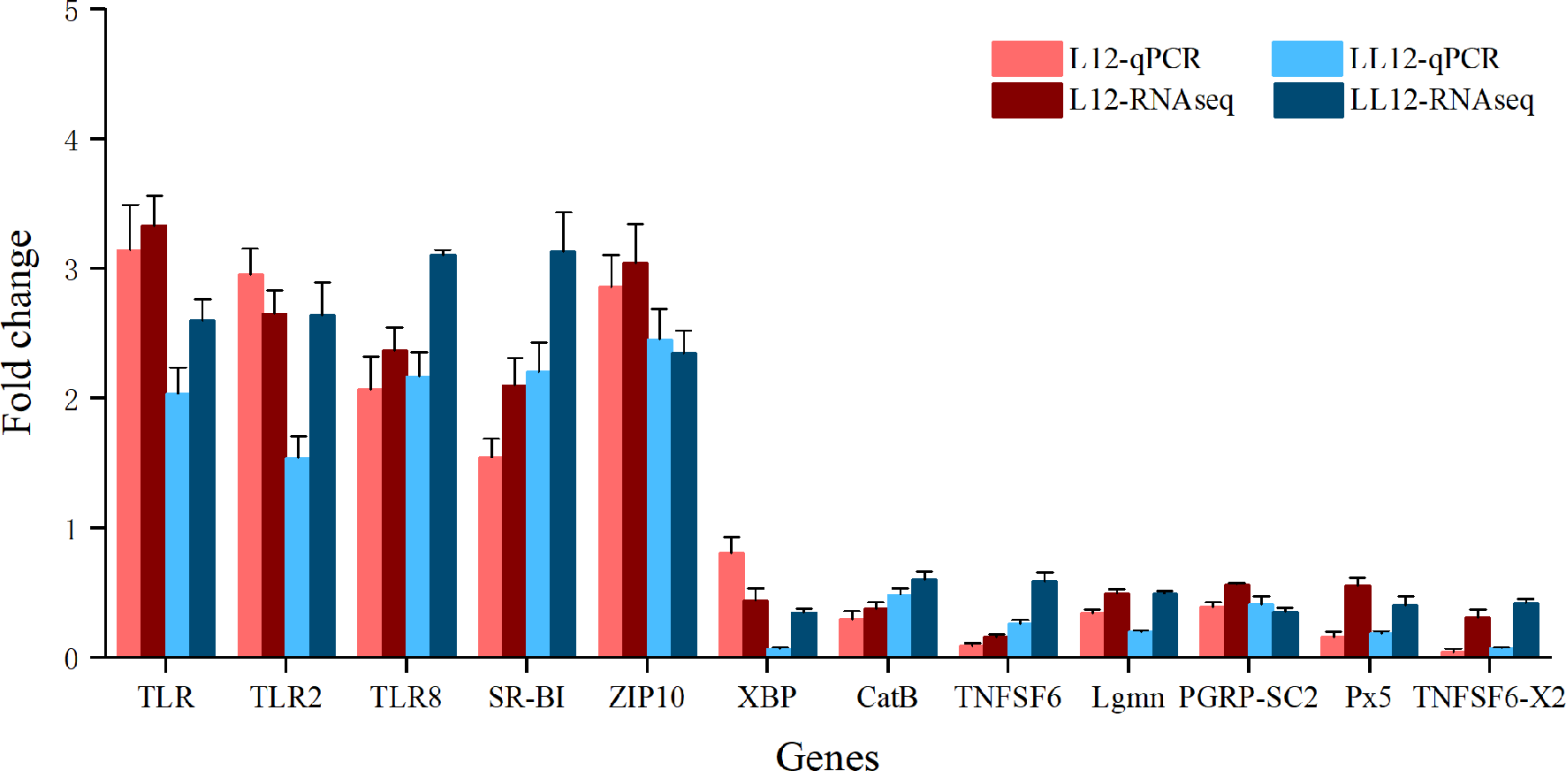
Quantitative RT-PCR validation of 12 genes that are differentially expressed after two successive infections. The x-axis is the gene name and the y-axis represents the fold change in gene expression compared to the control. Data are represented as mean ± SD for three biological replicates.

In order to evaluate the accuracy of the RNA-seq results, 12 genes including TLR, TLR2, TLR8, SR-BI, XBP, CatB, TNFSF6, Lgmn, ZIP10, PGRP-SC2, Px5 and TNFSF6-x2 from DEG libraries were analyzed by qRT-PCR. Fold changes were compared with the DEG analysis results. As shown in **Figure 6**, the expression trends of selective genes by qRT-PCR were consistent with the DEG analysis results, and the Spearman’s correlation was 0.875 (*p* value < 0.001), which indicated the reliability of the transcriptome data.

## Discussion

It was reported that immune priming was related to exposure dose and time post priming (2), only certain concentrations were able to induce a primed response, and low dose may lead to less obvious effects (23, 32). In the present study, the abalone were first injected with five different concentrations of *V. harveyi* diluents, which conferred the infected abalone with higher immune protection during subsequent re-exposure. We observed that different initial infection doses of *V. harveyi* provided different levels of protection; i.e., priming with the lowest initial infection dose provided the lowest immune protection, although the highest initial infection dose did not provide the highest immune protection (minimal immune protection was observed after priming with 1.42 × 10^3^ CFU mL^-1^ of *V. harveyi*, but 1.42 × 10^7^ CFU mL^-1^ did not offer maximum immune protection). Although enhanced immune protection has been found in many invertebrates, persistent immune protection time varied according to the invertebrate and pathogen examined (33–35). In mollusks, infection with *V. anguillarum* can offer *Chlamys farreri* immune protection for up to one week (17, 36). In *Biomphalaria glabrata*, immunological memory to *Schistosoma mansoni* was maintained for the rest of the animal’s lifespan (37, 38). In poly(I:C) primed *Crassostrea gigas*, resistance to Ostreid herpesvirus 1 (OsHV-1) could last for more than 5 months (39); and this protection was even transmitted to offspring (40, 41). In the current study, the time interval between priming and re-exposure was 15 days. Whether enhanced immune protection can last for longer time periods requires further research. However, in other species of abalone, it was reported that improved antibacterial response was induced within 25 days after injection of *V. fluvialis* in *H. discus hannai* (42). The appearance of a natural resistant population of the European abalone indicated that immune priming of abalone has strong plasticity, and functions within and across generations (26).

Previous studies have suggested that the formation of immune memory is related to the diversity of PRRs, the synergistic interactions between receptors and the expression level of receptors (43). In this study, PRRs in CL1 including perlucin 4, C1q domain containing protein 2, scavenger receptor class B-like protein, deleted in malignant brain tumors 1 protein-like, and 4 TLRs showed increased expression after re-exposure to *V. harveyi*. C1q domain containing proteins can bind to LPS, PGN, polyI:C, beta-glucan, mannan, and yeast-glycan, giving them a broader bacterial agglutinating spectrum, and powerful function in the recognition strategy. In addition, they can act as opsonins to enhance phagocytic activity (44–46). Scavenger receptors (SRs) are major endocytic receptors that can bind Gram-negative and Gram-positive bacteria to promote hemocyte phagocytosis (47, 48). Perlucin is a typical C-type lectin, which can directly agglutinate bacterial pathogens to restrict their spread in plasma, and is involved in regulating the innate immunity by regulating phagocytosis and AMP expression (49). Toll and toll-like receptors are involved in pathogen recognition in plants, invertebrates and vertebrates (50). In Drosophila, the toll pathway was reported to regulate hemocyte proliferation (51), antimicrobial peptide expression (52), and initiate a systemic response in which hemocytes are mobilized and activated (53); more importantly, specific immune priming requires the toll pathway (19). In mollusks, a number of TLRs have been identified in various species, including hard clam (54), mussels (55, 56) and oysters (57, 58). TLRs were highly expressed in molluscan hemocytes and the TLR pathway was suggested to play a central role in initiating the cellular response to infection (59). It has been reported that TLR signaling is involved in enhanced immune protection of oysters against pathogen re-infection (60). In this study, the increased expression of PRRs suggested their contribution to improved immune protection during the re-exposure of abalone. Moreover, synergistic interactions may exist between different PRRs to enhance pathogen clearance.

In invertebrates, cellular immune responses are performed by hemocytes. Phagocytosis is the core defense mechanism for hemocytes to eliminate external invaders (14, 61). Many studies have shown that phagocytosis is necessary for the evaluation of immune memory in invertebrates. Studies have reported that specific primed immune responses of Drosophila (19) and silkworm (18, 62) were dependent on phagocytes. In mollusks, the total hemocyte count and phagocytic activity were increased after secondary infection in snails (63), scallop (36) and oysters (20, 64). In the present study, we demonstrated the importance of phagocytosis from a molecular perspective, as some genes in CL1 involved in lysosome, phagosome, endocytosis and Fc gamma R-mediated phagocytosis had higher expression levels in secondary infection. In addition, among the specifically expressed genes, CL7 contained more gene annotations to phagocytosis-related pathways than CL6. In oyster, sequencing results also showed that Fc gamma-mediated phagocytosis and ubiquitin-mediated proteolysis were significantly enriched, and phagocytosis was suggested to have a key role in second immune protection (60). In addition, the activation of immune-related pathways in CL4 also plays an important role in pathogen clearance (**Supplementary File 3**). With the exception of the classical immune-related pathway, some genes involved in the calcium signaling pathway were also significantly up-regulated in the present study. Calcium signaling is critical for diverse biological processes including fertilization, differentiation, proliferation and gene transcription (65). Calcium signaling also plays a vital role in immune function (66). Weavers et al. (67) demonstrated that calcium bursts mediate molecular memory generated by corpse engulfment by Drosophila macrophages. In addition, C1q domain containing proteins, scavenger receptors and perlucin functionally promote phagocytosis. Thus, it can be speculated that phagocytosis plays an important role in preventing re-infection of the same pathogen in abalone.

Effector factors are the most basic molecules, and are the main agents involved in the elimination of pathogens. Of these factors, antimicrobial peptides (AMPs) constitute the first line of host defense against pathogen infection, and are crucial components of the innate immune system in mollusks (68). To date, several types of AMPs have been identified and characterized from mollusks, including mytilins, myticins, mytimycins, mytimacins, defensins, big defensins, histones, lysozymes, abhisin and molluscidin (69–75). In marine mollusks, AMPs can bind the bacterial membrane directly and kill the invading bacterial pathogens by membrane disruption (76). In this study, only lysozyme in CL1 was found to be notably up-regulated in secondary infection. This may have been due to the sampling time points, as not all AMPs transcripts were increased 12 h after pathogen infection (77–79). However, AMPs are only effectors of the immune response and have a spectrum of antimicrobial characteristics, which can be induced indiscriminately, and the AMP transcripts quickly return to the baseline state after infection (19). Therefore, the AMPs are considered unable to establish a primed response (1, 18). We also found that a range of immune effectors associated with detoxification and antioxidant stress in CL1 were significantly increased during re-infection, including cytochrome P450 1B1-like, glutathione S-transferase 6 and small heat shock protein (80–82). Our sequencing results showed that the enhanced immune protection after secondary infection was not only due to the role of AMPs, but also involves the synergistic effect of multiple immune effectors.

In mammals, there is increasing evidence that trained immunity involves metabolic regulation such as glycolysis, oxidative phosphorylation, fatty acid and amino acid metabolism, which endows innate immune cells with the ability to respond more strongly to a second stimulus (83). Glycolysis, an alternative form of glucose metabolism, can produce ATP faster and it is thought to play a key role in immunity (84). Induction of glycolysis has recently been shown to be crucial for the initiation of trained immunity in human volunteers after BCG vaccination (85). Epigenetic modification also plays an important role in the establishment of innate immune memory. Several metabolites of glycolysis and the TCA cycle are also co-factors for epigenetic enzymes (86). Fatty acids can activate innate immune pathways, amino acids are the basic chemical building blocks during biogenesis, lipid metabolism and amino acid metabolism, and were proved to be necessary for the induction of innate immune memory (83, 87). In this study, we also found that many genes involved in glycolysis, fatty acid and amino acid metabolism were significantly up-regulated in CL1 (**Supplementary File 4**), and more genes were found in CL7 than in CL6 (**Figure 5**; **Supplementary File 5**). This suggested that metabolic pathways also played a role in abalone immune priming. In addition, it was found that innate memory in macrophages could polarize other neighboring cells in ways that drive antibacterial, Th17 and M1 responses (88). This suggested that intercellular signal transduction played an important role in bacterial clearance in re-infections. The results in **Table 3** and **Figure 5** also demonstrated this, as more genes were annotated to signal transduction in CL7.

In summary, in the present study we demonstrated that an infection enhanced abalone immunity to secondary infection with the same pathogen, although this protection was not linearly correlated with the initial infection dose. Comparative transcriptome analysis has improved our understanding of the mechanism of enhanced immune protection in abalone. Increased immunity in abalone was due to the synergistic effect of the recognition of a variety of pattern recognition receptors, phagocytosis of hemocytes, detoxification and anti-oxidation of immune effectors, the enhancement of metabolism, and so on. The study on the mechanism of immune protection enhancement of abalone carried out in this study will enrich the content of invertebrate immunology, and contribute to a deeper understanding of the diversity of invertebrate immune priming mechanisms and the evolutionary process of the invertebrate immune system.

## Data Availability Statement

The datasets generated for this study can be found in the NCBI Short Read Archive database under the accession ID SRR13931757-SRR13931765.

## Ethics Statement

The animal study was reviewed and approved by the Animal Care and Ethics Committee of South China Sea Fisheries Research Institute, Chinese Academy of Fishery Sciences.

## Author Contributions

TY and LY conceived, designed the experiment, and wrote the manuscript. TY and JL performed the experiments. CB and ZX analyzed the data. All authors contributed to the article and approved the submitted version.

## Funding

This work was supported by grants from the National Key R&D Program of China (2019YFD0900105), the Science and Technology Planning Project of Guangzhou (202002030488), the China Agriculture Research System of MOF and MARA, the Central Public-interest Scientific Institution Basal Research Fund, CAFS (2020TD42 and 2021SD05), the Science and Technology Planning Project of Jieyang (2019029 and sxm029), the Professorial and Doctoral Scientific Research Foundation of Huizhou University (2020JB065), the Shellfish and Large Algae Industry Innovation Team Project of Guangdong Province (2020KJ146) and the Guangdong Rural Revitalization Strategy Special Funds (Fishery Industry Development) (YueCaiNong[2020]4).

## Conflict of Interest

The authors declare that the research was conducted in the absence of any commercial or financial relationships that could be construed as a potential conflict of interest.
